# A systematic review of the outcome data supporting the Healthy Living Pharmacy concept and lessons from its implementation

**DOI:** 10.1371/journal.pone.0213607

**Published:** 2019-03-12

**Authors:** Zachariah Jamal Nazar, Hamde Nazar, Simon White, Paul Rutter

**Affiliations:** 1 College of Pharmacy, Qatar University, Doha, Qatar; 2 School of Pharmacy, Newcastle University, Newcastle upon Tyne, United Kingdom; 3 School of Pharmacy, Keele University, Keele, United Kingdom; 4 School of Pharmacy and Biomedical Science, University of Portsmouth, Portsmouth, United Kingdom; Drake University College of Pharmacy and Heath Sciences, UNITED STATES

## Abstract

**Background:**

The Healthy Living Pharmacy (HLP) project, launched in England, UK in 2009 was a novel approach of introducing public health services within community pharmacy to tackle local health inequalities. A national roll-out followed a reported successful pilot; subsequent local evaluations ensued.

**Objectives:**

To summarise reported outcomes and investigate contextual factors that indicate the presence, absence and maturity of implementation determinants, thus offering useful lessons to stakeholders in implementing future initiatives to achieve successful outcomes.

**Methods:**

A systematic review was conducted to identify all publications reporting on the HLP project. All HLP articles and conference abstracts were considered for inclusion and were assessed for methodological quality. The Consolidated Framework for Implementation Research (CFIR) was utilised to identify potential implementation determinants reported. Each article was then analysed to identify reported economic, humanistic or clinical outcomes.

**Results:**

The review included six peer-reviewed journal articles and 12 conference abstracts. Joanna Briggs Institute Qualitative Assessment and Review Instrument indicated deficiencies in methodological quality. Through adoption of the CFIR framework, the implementation determinants relevant to the implementation of HLP into community pharmacy were identified. A resonating issue emerged in that the absence of adopting an evidence-based implementation process limited the ability to capture meaningful outcome data. This resulted in a lack of evidence to support sustainability and the failure to address many of the well cited barriers, e.g. lack of awareness amongst patients, public and other healthcare professionals, and weak support for future investment in resource for training and dissemination.

**Conclusions:**

Healthcare systems are increasingly called on to adopt evidence‐based interventions that improve quality, control costs, and maximize value, thus offering opportunity to accelerate the implementation of clinical pharmacy services and programs aimed at improving patient care. Interventions, such as the HLP project require focused efforts on implementation and evaluation of those implementation efforts to produce effective and lasting changes in complex health care systems.

## Introduction

The 2008 White Paper: *Pharmacy in England: building on strengths, delivering the future,[1]* described the role community pharmacy could play in supporting public health through becoming healthy living centres. Recommendations were made to increase pharmacy’s contribution to promoting better health, prevention and early detection of disease and managing patients with long-term conditions. The development of the concept of the “healthy living centres” was commissioned by the Department of Health (DoH) to Portsmouth Primary Care Trust in 2009. The project was named ‘The Healthy Living Pharmacy (HLP) project’.

The HLP model is based on a tiered framework that is designed to quality assure the delivery of specific services to meet local public health demands.[2] It consists of three levels of advancing service provision, each level underpinned by several key principles. Firstly, the services are tailored to local health needs with the aim of reducing health inequalities by improving health and wellbeing outcomes in their communities. Secondly, a HLP builds on existing core pharmacy services (Essential and Advanced) with a series of Enhanced Services (Table 1).[3]

**Table 1:** NHS (Pharmaceutical Services) Regulations 2005 in England. [3].

**A.Essential services and clinical governance:** Essential services are provided by all pharmacy contractors and are commissioned by NHS England. Examples include dispensing, disposal of unwanted medicines and public health. Clinical governance includes patient safety incident reporting, an information governance program, patient and public involvement in service delivery and premises requirements.
**B. Advanced services:** These are provided by all contractors once accreditation requirements have been met and are commissioned by NHS England. Examples include Medicines Use Reviews and the New Medicine Service.
**C. Locally commissioned enhanced services**: These are commissioned by Local Authorities, Clinical Commissioning Groups and NHS England in response to the needs of the local population.

Finally, the delivery of services is supported by three enablers: workforce development, with the introduction of Health Living Champions (HLCs); premises fit for purpose, including a space to facilitate private consultations within the pharmacy and a dedicated health promotion area; and, local stakeholder engagement, including local General Practitioners (family doctors) and members of the public.

This community pharmacy initiative attempted to address a number of recognised implementation determinants established in the related literature. The introduction of a dedicated health promotion area and a space for private consultations, as well as stakeholder engagement aimed to enhance poor public understanding of the role of pharmacists[4] and the professional isolation of community pharmacists,[5] and address the lack of a space for private consultations.[4] These factors have been cited as barriers to the successful implementation and embedding of community pharmacy initiatives in the UK[6, 7] and in the US, among other countries, for example in introducing pharmaceutical care in community pharmacy.[8] A recent independent review commissioned by the National Health Service (NHS) in England also lists these factors, amongst others, that are recognised as barriers to community pharmacy providing clinical services.[9] The involvement and training of non-pharmacist team members attempted to facilitate the effective delegation of responsibilities to pharmacy support staff and enhance the skill-mix as advocated in previous studies.[10, 11] Prior to this, commissioners had continued to focus on remuneration as the single most important factor in introducing innovative services into community pharmacy with little consideration to other factors.[12] This frequently resulted in poor uptake of the service and consequent discontinuation of commissioning.[13–16]

A model for HLP was launched in December 2009 through publication of a local HLP prospectus. This was informed by a systematic review of the evidence for the role of community pharmacy in a range of services to support their inclusion in the HLP initiative.[2] Community pharmacies were invited to apply to be Level 1 HLPs, which required participation in the following services: wellbeing and self-care, including active health promotion campaigns, optimising medicines including delivering targeted respiratory Medicines Usage Reviews (MURs); accredited pharmacists undertaking structured adherence-centred reviews with patients on multiple medicines, particularly those receiving medicines for long-term conditions) and providing two enhanced services, one of which had to include smoking cessation. The full accreditation criteria are detailed in S1 Table.

To meet the ‘workforce development’ enabler requirement, at least one non-pharmacist member of the pharmacy team was required to become a qualified HLC by achieving an accredited qualification in understanding health improvement. This was intended to be an individual with an interest in public health and a commitment to the HLP concept.

A local evaluation of the HLP project demonstrated an increased uptake of community pharmacy services in Portsmouth, which informed the roll-out of a national pathfinder programme across England[17, 18]. The HLP models in these pathfinder sites were similar to the Portsmouth model, but with local variation in the services that were commissioned and the support that was provided to HLPs. More recently, the National Health Service in England introduced a Quality Payments Scheme, which provided a financial incentive for community pharmacies to become accredited as HLPs, among other quality initiatives.[19]

As a result of the national roll-out, there is now a small but growing body of literature reporting on the implementation and outcome data of HLP, but a review of these individual studies to examine trends in challenges to implementation and the quantitative service data of HLP has not yet been reported. It has been argued that in recognising these determinants, intervention benefits can be optimised, sustainability of the intervention can be prolonged, and dissemination of findings into other contexts can be enhanced.[20] Moreover, in reviewing the reported outcome data, it will be possible to identify, if any, the economic, humanistic and clinical outcomes.

The barriers and facilitators of introducing new services into community pharmacy has been reported in the literature,[12, 21, 22] however, implementation science research adopts the use of theoretical models allowing researchers to systematically collect, analyse and interpret appropriate data in evaluating the implementation of innovation. It is advocated that this approach within healthcare, affords recognition of contextual and processual dimensions commonly linked to failures of implementing innovation.[23, 24]

This review aims to summarise the reported outcomes of individual HLP evaluations and investigate contextual factors included in those evaluations that indicate the presence, absence and maturity of implementation determinants.[23, 24] This will offer useful lessons to stakeholders in the implementation of future HLP initiatives towards achieving successful outcomes.

## Methods

### Data sources and search strategy

**Fig 1:**
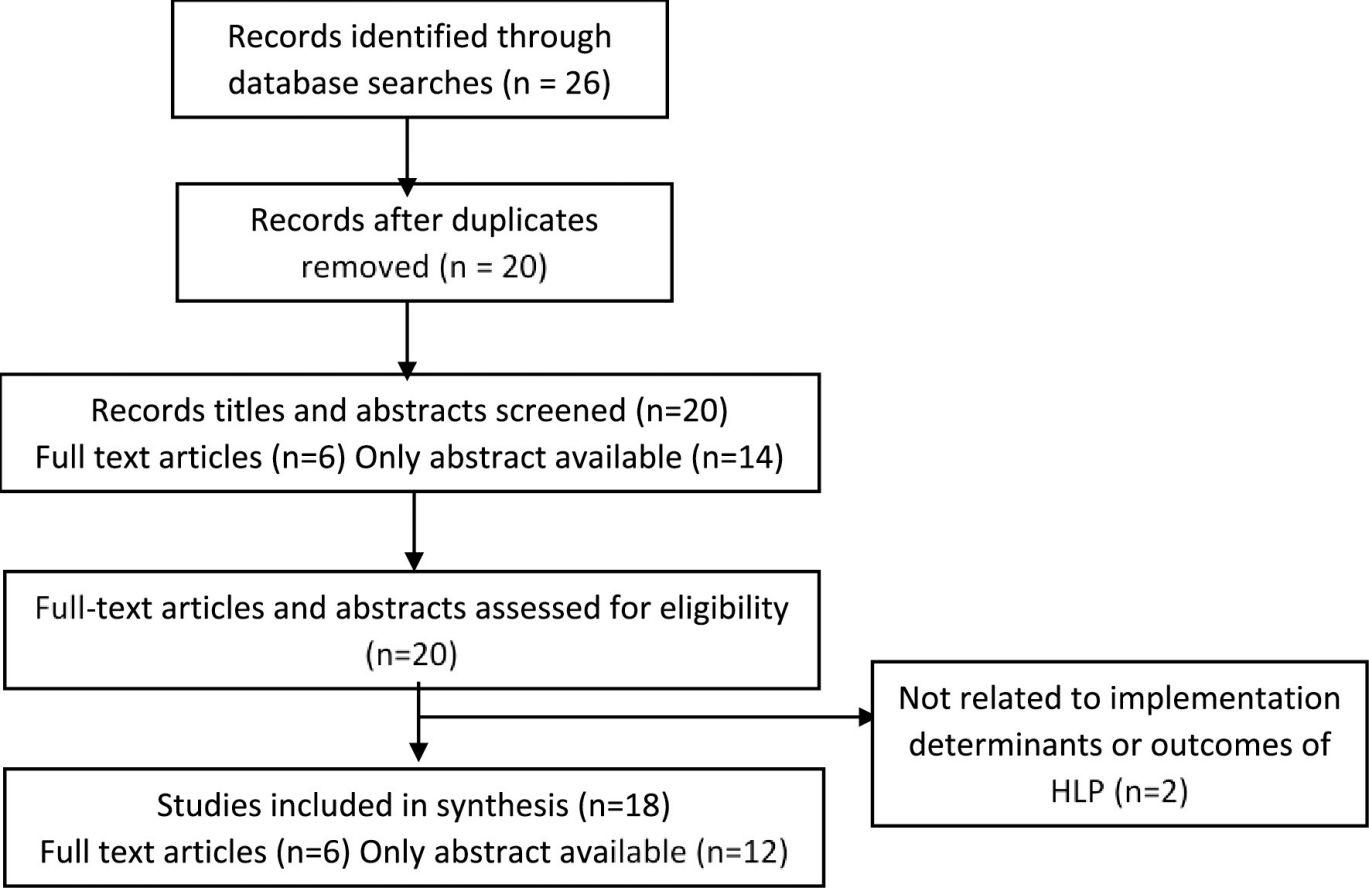
Study selection process (PRISMA flow diagram).

This systematic review is reported in accordance with the Preferred Reporting Items for Systematic Reviews and Meta-Analyses (PRISMA) guidelines. The study selection process (PRISMA flow diagram) is presented in Fig 1 and the completed 27-item checklist is included in S2 Table. The five-step principle of conducting a systematic review as described by Khan et al. was adopted, which includes defining the research question, identifying the literature, quality assessment of the selected literature, summarising the findings and interpreting the evidence.[25]

The SPICE (setting, perspective/population, intervention, comparator, evaluation) framework[26] was employed by one research team member [ZN] to design the research strategy and included a search of MeSH (medical subject headings) terms and title/abstract terms (TIAB) [Table 2]. The full electronic search strategy used to identify studies, including all search terms and limits for one database (MEDLINE) is included as S3 Table. Four electronic databases (Google Scholar, Web of Science, MEDLINE and PubMed) were searched from January 1^st^ 2000 to 31^st^ March 2018. Electronic searches were supplemented with hand citation searches and reference list review of eligible studies. In addition, conference proceedings reported in the International Journal of Pharmacy Practice (IJPP), which includes two of the most recognised international pharmacy practice conferences and are not indexed in the major databases) and International Social Pharmacy Workshop (ISPW) were reviewed. A conference abstract was only considered for inclusion for review in this study where a full paper on that same study was not available. A review of titles, abstracts and full texts was carried out independently by two authors [ZN & HN]. Any disagreements over included articles were discussed with a third reviewer to mitigate against potential bias.

**Table 2:** Generalised search terms used informed by the SPICE framework. (No comparator groups were identified)[26].

Setting	Perspective/Population	Intervention	Evaluation
Community	Pharmacy	Healthy living pharmacy	Outcome
Primary care	Community pharmacy	Healthy centre	Perception
		Public health	Feedback
		Pharmaceutical care	Barrier
		Healthy living champion	Facilitator
		Champion	Economic
			Clinical

Any quantitative or qualitative studies published in English that reported on data on the HLP initiative were included; no date restriction was set and articles were eligible for inclusion regardless of the year published.

### Data extraction and quality assessment

Two investigators (ZN and HN) independently evaluated the full-text of the identified articles to determine whether they met the inclusion criteria, which were that papers must directly concern implementation of the HLP initiative or provide HLP outcome data. Any disagreements over included articles were discussed and resolved through consensus between both investigators and, where necessary, a third reviewer was consulted.

An Excel spreadsheet was created for the purposes of data extraction, which contained a row for each included article and columns to describe the studies and classify the extracted data related to implementation determinants and reported outcomes. After identifying the included studies, the two researchers reviewed each of the selected studies and assessed the methodological quality of qualitative studies using the Joanna Briggs Institute Qualitative Assessment and Review Instrument (JBI-QARI).[27] The Instrument includes criteria such as: the presence of congruity between the different parts of the qualitative research; adequate representation of all participants, and assesses the consistency of the conclusion with the analysis of the results. This critical appraisal tool consists of 10 questions that address the possibility of flaws in design, conduct, or analysis. For each question a rating of ‘yes’, ‘no’, ‘unclear’, or ‘not applicable’ was allocated. The number of ‘yes’ responses for each article was totalled to give a score out of 10. Once completed, the two researchers met with a third investigator to discuss ratings and come to agreement on discrepancies. One point was awarded for each ‘yes’ and a total score was obtained for each study. (The quality assessment is summarised in S2 Table)

### Coding and synthesis of the literature

Many theories, models and frameworks have been developed to explain and support the implementation of interventions in healthcare. Examples of some of the more commonly used frameworks are the Consolidated Framework for Implementation Research (CFIR),[28] the Active Implementation Frameworks (AIFs),[29] and the RE‐AIM evaluation framework.[30] Uniquely, the CFIR is based on a critical review of the literature on professional services provided in community pharmacies and is the most widely cited and used in the pharmacy practice literature.[31] To synthesise the implementation determinants from the literature, the CFIR was chosen because of its ability to situate potential implementation determinants across a range of domains.[28] The CFIR provides a framework that specifies a list of implementation determinants within general domains, which are believed to influence implementation based on the strength of their support in the healthcare literature. The CFIR is composed of five major domains: i) intervention characteristics, ii) outer setting, iii) inner setting, iv) characteristics of the individuals involved in implementation, and v) the process by which implementation is accomplished.

Two investigators (ZN and HN) read the included articles multiple times and then independently undertook the coding through developing a list of reported implementation determinants to the implementation process of HLP. The two investigators then independently catalogued the themes that emerged according to the CFIR framework constructs. The two investigators then met to discuss and reconcile codes, identify emergent themes, and resolve discrepancies through consensus. It should be noted that this involved interpretation of whether there were findings relevant to a CFIR domain, since none of the studies adopted an implementation framework in reporting their findings. Each article was then independently analysed by the two investigators to identify reported outcomes, which were coded into either economic, humanistic or clinical.

## Findings

The literature search resulted in twenty publications reporting on the implementation of the HLP or providing outcome data, which were published between 2013 and 2017 (Table 3); six peer-reviewed journal articles and fourteen conference abstracts. Studies were predominantly qualitative, involving case studies and interviews and/or surveys of the perceived impacts, success factors, barriers, or satisfaction among different stakeholder groups. Sixteen of these publications included reports of implementation determinants (Table 3).[17, 32–46] Four publications,[17, 32–34] reported on both implementation determinants as well as outcomes of HLP involvement; and two publications reported solely on various outcomes of HLP involvement.[47, 48] Two publications reported on the content of HLC training and therefore did not fall into the criteria since.[49, 50] Identified articles were placed in chronological order starting from 2013 to 2017.

**Table 3:** Studies reviewed, the type of study and CFIR domains represented in each.

		Consolidated Framework for Implementation Research domain				
First author (year)	Type of study	Intervention characteristics	Outer setting	Inner setting	Individual characteristics	Process
**Kennington et al. (2013)**[47]	Qualitative; service provider survey	X	X			
**Nazar et al.** **(2013)**[35]	Qualitative; pharmacy staff interviews		X	X	X	X
**Brown et al.** **(2014)**[17]	Mixed methods; service outcome data & pharmacy staff interviews	X	X	X	X	X
**Rutter et al.** **(2014)**[39]	Qualitative; pharmacy staff interviews	X	X		X	
**White et al.** **(2014)**[40]	Qualitative; pharmacy staff interviews		X	X	X	X
**Donovan et al. (2014)**[41]	Qualitative; pharmacy staff interviews			X	X	
**Patel et al. (2014)**[46]	Qualitative: pharmacy staff interviews and surveys		X	X	X	
**Shevket et al.** **(2015)**[43]	Qualitative; pharmacy staff interviews	X	X	X	X	
**Firth et al. (2015)**[44]	Mixed methods; service outcome data & pharmacy staff interviews	X	X	X		X
**Donovan et al. (2015)**[42]	Mixed methods; service outcome data & pharmacy staff interviews	X	X	X	X	
**Kayyali et al. (2016)**[49]	Quantitative; HLC training survey	X		X		
**Nazar et al.** **(2016)**[36]	Qualitative; Pharmacy staff survey		X			X
**Cooper et al. (2017)**[45]	Qualitative; pharmacy staff focus groups and interviews		X	X	X	X

The studies were conducted across England, including locations in the north, south, and central areas of England as well as the capital, London. Studies where the focus was investigating community pharmacy staff views and perceptions were not limited to an individual pharmacy, but rather included multiple pharmacies within a geographic location. Data was gathered from individuals representing pharmacies located in different areas of deprivation; city centre, urban and rural pharmacies; and in different types of community pharmacies from the single independent to the large multiples. Where reported on, authors stated that location, area of deprivation and type of pharmacy did not reveal distinguishable trends. Similarly, there was no mention that size of the pharmacy, in relation to prescription volume or workforce number, correlated to contrasting findings.

All but one study included qualitative methodologies and were subjected to appraisal using the JBI-QARI, which revealed that deficient methodological quality was present in all of the published studies. Scores ranged from 2 to 8, with five studies receiving a score of 8 and an overall median score of 5. This was most notably recognised through the lack of detail relating to the philosophical or theoretical perspective of the research; consequently, in much of the research it was not possible to identify congruence with the methodological approach adopted. Furthermore, since many of the studies were conducted by pharmacists, it would have been appropriate for a statement to have been included that located the researcher culturally and the potential influence the researcher had on the research, including the steps taken to limit this. To evidence transferability and dependability, four studies provided rich and contextualised descriptions of the research context;[17, 39, 42, 44] however the majority of the studies did not describe the interview context, interview duration, or examples of interview questions. Only four studies described how interview questions were developed,[17, 37, 41, 44] and only two studies described how they were pilot tested.[41, 42]

The findings below are presented by each of their five CFIR domains (i.e. intervention characteristics, outer setting, inner setting, individual characteristics, and process); within each of these domains there are several constructs (see http://cfirguide.org/ for a description of each construct). The X indicates where a construct within one of the five domains was implicated as an implementation determinant and may have acted as a barrier or an enabler in reporting the implementation of HLP.

Table 4 provides details of the constructs identified through analysis of the published literature found to be relevant to the implementation of the HLP and Table 5 presents a summary of the reported outcomes from the HLP literature, categorised into economic, clinical or humanistic characteristics.

**Table 4:** CFIR Domains: Implementation characteristics relevant to the HLP model.

Intervention characteristic	
Relative advantage	Staff recognised the HLP model to provide a more proactive, supportive and effective approach to service delivery. [35, 39, 40, 42, 44] Staff perceived an opportunity for personal betterment in HLP involvement. [34–36, 39, 40, 45]
Evidence strength and quality	Commissioners viewed HLPs as an important delivery mechanism for public health services.[47] Contractors and employers felt that service commissioning was not based on local needs.[47, 49] Commissioners raised concerns about the lack of evidence base for the services included in the HLP model.[47]
Adaptability	Staff recognised that many of the services included in the HLP model were already delivered in the pharmacy and were integrated into existing workflow. [35] [41]
Cost	Pharmacists did report their concerns regarding the setting up and delivery cost. [42, 44, 45]
Outer setting	
External policies and incentives	Problems with receiving remuneration for service provision.[35] Lack of awareness of both clients and health care professionals locally.[34, 35, 39, 40, 42–45] Lack of nationally agreed accreditation criteria.[35, 47]
Cosmopolitanism	Difficulties in establishing new relationships with local organisations for patient referrals and synchronised health promotion activities.[40, 42, 43, 45]
Patient needs and resources	Patient access and acceptability of services delivered through the HLP project were positive.[32–34, 42, 43] However, access for hard to reach patients was not considered e.g. homeless and housebound patients. [45]
Inner setting	
Structural characteristics	An additional computer terminal wired to the internet was identified as an enabler to support patients in signposting and providing further advice. [17] The lack of available space led to challenges in creating suitable area for health promotion activities.[45]
Implementation climate	*Relative priority and compatibility*: Tension between employers and staff around prioritising commercial aspects of community pharmacy with the HLC role.[35, 40] *Compatibility*: Pharmacists recognised the HLC role to be crucial in furthering progress with HLP accreditation and supporting the delivery of services.[17, 41, 45] *Learning climate*: Staff were motivated to undertake new learning and advance their role to benefit patient care.[35, 36, 39, 43, 49]
Readiness for implementation	*Available resources*: The convenience, time and cost investment of training staff posed an obstacle to undertake the HLC course. [17, 49] *Access to knowledge and information*: HLCs used their network for peer-learning and obtaining advice and information.[36, 38]^.^
Characteristics of individuals	
Knowledge and beliefs about the intervention	Staff felt that the HLP model provided greater job satisfaction and enhanced their professional development and confidence.[17, 36, 39, 45] Staff reacted positively to the HLP concept and the services included.[39, 44, 45]
Other personal attributes	Staff were enthusiastic and motivated to explore opportunities to do different activities afforded by the HLP model.[35, 39, 40, 45, 49]
Individual identification with organisation	Staff perceived the HLP model to be a natural extension of their current roles and believed that the model fitted the ethos of community pharmacy.[17, 39, 41, 45]
Process	
Engaging	*Champions*: HLCs supported staff with customer engagement.[17, 36, 42, 45] *External change agents*: Little work by commissioners to raise local awareness and encourage involvement of local health and social service.[40, 45] *External change agents*: Commissioners demonstrated a poor understanding of the HLP framework. [42, 44]
Planning	The absence of a facility for disseminating and sharing HLP information was recognised as a barrier.[36, 42, 45]
Reflecting and evaluating	The lack of communication regarding local and national progress with HLP propagated feelings of isolation.[17, 36, 45]

**Table 5:** Summary of reported HLP outcomes.

Reported outcomes	
Economic	Evidence that service users would have gone to their family doctors, or would have done nothing if they had not accessed the community pharmacy service. [32, 33]
Clinical	The smoking cessation programme run in HLP accredited pharmacies supported a larger number of patients to reach a 12-week quits [17, 48].
Humanistic	The public reported HLP accredited pharmacies as a new option to access health and well-being services, which were convenient and timely. [17, 32, 34]

## Discussion

Our appraisal of the published research has highlighted that the current body of literature has insufficiently addressed the possibility of bias in its design, conduct and analysis. Furthermore, a key review article which contributed to the evidence base for the national role-out of HLP contained a lack of both theoretical underpinnings and analysis transparency.[51] It has been advocated that a formative, theory-based approach be taken in conducting evaluations of initiatives in pharmacy practice.[52, 53] This includes recommendations that evaluations account for the iterative nature of health care improvement work and are undertaken prospectively, generating learning applicable to ongoing improvement efforts and enabling midcourse adjustment to the initiative. Moreover, it has been recommended that such initiatives are resourced to include a trained programme evaluation researcher to conduct this work.[54, 55] The findings relating to the implementation determinants are discussed with reference to the relevant constructs within each domain.

### Intervention characteristics of the CFIR

Relative advantage, evidence strength and quality, cost and design quality and packaging were found to be relevant implementation determinants for the HLP model in community pharmacy (Table 4).The community pharmacy stakeholders interviewed in the reported studies perceived that HLP involvement would offer benefits to the public as well as providing opportunities for professional development. It is important to note, however, that the majority of the reported studies were conducted with pharmacy staff employed in pharmacies working towards or which had achieved HLP accreditation; very few reports included the perceptions of staff employed in pharmacies that were not working towards HLP accreditation. It can therefore be argued that the sample populations included in the studies may have been biased and the relative advantage, which referred to pharmacy staff perceiving advantages of implementing the HLP model, may not be perceptions held by the wider pharmacy staff population. This observation has been recorded in related literature which recognises the concept of ‘Leading Edge Practitioners’ (LEPs).[56] LEPs have been identified as enablers of successfully implementing innovation, through adopting a proactive approach to integrating new methods of working, focussing on staff development, networking with peers and establishing channels to enhance their influence. LEPs have also been recognised as individuals who are more likely to participate in evaluative research to contribute their experiences to the development of professional initiatives. It is unclear from the published studies whether these reported perceptions stemmed from local communications of HLP developments or whether reports of HLP in the national press had propagated these positive perceptions towards the initiative.

In many of the studies, it was found that achieving the HLP accreditation criteria had not been perceived as unduly challenging, since a large number of community pharmacies had already been offering the majority of the services that the HLP initiative had intended to promote. In contrast, in those pharmacies which had not previously offered these services, staff reported their concerns of the cost of implementation as a barrier, which delayed and in some cases deterred their involvement. Although it was not stipulated what these costs referred to, further analysis of the various implementation resources suggested that the only visible costs related to costs incurred by sending staff on training courses and forecasted costs of rearranging workflow in the pharmacy to manage the potential demand for services.

The few studies which focussed on the views of commissioners described their belief that the HLP initiative could pose an important delivery mechanism for commissioning public health services. However, they shared their scepticism over the evidence base for the services encompassed in the initiative and discussed the need to assess the design of the initiative according to local needs and resources.

The current data indicates that the HLP initiative provides a framework to structure and motivate public health services in community pharmacy. However, concern has been expressed about the evidence for the effectiveness of these services and that the extra cost may not warrant the training and resourcing for services already provided within community pharmacy outside the HLP model.[45, 47] Given that studies until now do not provide a great deal of outcome data to assess the effectiveness of HLP with regards to economic, clinical and humanistic outcomes, the expressed concerns may potentially remain unaddressed and limit the sustainability of the HLP model.

### Outer setting of the CFIR

Generally, the outer setting includes the economic, political, and social context within which an organization resides;[28] Table 4 provides an overview of the relevant *outer setting* constructs observed in the HLP literature.

The *outer setting* constructs that appeared to be most relevant were ‘external policies and incentives’, ‘cosmopolitanism’, and ‘patient needs and resources’.

The earlier studies reported community pharmacy staff frustrations with the cumbersome process of claiming reimbursement for the delivery of HLP services.[17, 35] Subsequently, the paper-based method that required postal submission to the commissioners at the end of each calendar month was replaced with PharmOutcomes, a flexible, integrated web-based system that facilitates recording and analysing service details and submission of data to the commissioners.

The *outer setting* construct that appeared to be reported most frequently was that of the wider awareness of the HLP initiative. It was reported in the majority of studies that community pharmacy customers, the public and local health care professionals appeared to have had little, if any, awareness of the initiative and that this was perceived to contribute to challenges in recruitment to HLP services and developing relationships with local organisations affiliated to HLP initiatives. This finding is consistent with a recent Royal Pharmaceutical Society (RPS) study, which includes poor public understanding of the role of pharmacists and community pharmacy being professionally isolated and marginalised within the National Health System (NHS) as barriers to implementing innovation in community pharmacy.[57] Similarly, this finding reflects reported barriers cited in the wider related literature.[8]

The lack of consistent accreditation criteria across different geographic commissioning regions many well have been a factor, which deterred a large national multiple pharmacy chain from supporting the HLP initiative.[44] It was reported that without a set of consistent accreditation criteria, the level of investment could not be determined; this has since been addressed with the embedding of the criteria in the national community pharmacy contractual framework.[58]

Studies in which patient feedback was collected indicated that patients who had accessed HLP services reported positive experiences with regards to receiving the service through community pharmacy, which is consistent with the related literature concerning patient experience with community pharmacy services.[4] However, it was reported that although the HLP initiative targeted local health inequalities, there was little attention directed towards homeless and housebound patients who are often in the most need of additional services.

The poor public and patient awareness of the roles and capabilities of pharmacists has been documented in a systematic review,[4] which reported that consumers may regard pharmacists as appropriate providers of public health services but often lacked confidence in their ability. Those consumers who had accessed such services in community pharmacy reported good satisfaction with the experience and the review recommended that further training and opportunity should be provided to pharmacists to increase public confidence and awareness. Despite this training and opportunity being part of the HLP model, patient perceptions broadly appear to be similar to those captured by Eades *et al*.[4] This review offers some insight on this issue, in that the time to achieve change in perception is lengthy and research may not start to capture this until there has been a sustained delivery of public health services through HLP.

### Inner setting of the CFIR

Aspects of the organisational culture, structure and politics that reflects receptiveness to change and new ways of working in the organisation are categorised as inner settings.[28] Table 4 provides an overview of the relevant *inner setting* constructs observed in the HLP literature.

*Structural characteristics* did not appear as prevalent implementation determinants; where these were cited it was generally by staff employed in smaller pharmacies, where issues such as inadequate available space to support health promotion activities were reported.

The implementation climate refers to both positive and negative implementation determinants. The compatibility of the HLC role seemed to vary in the separate reports; since the role was identified as a key enabler for HLP accreditation in a large number of pharmacies through contributing to staff training and championing the various HLP services. In contrast, in others, a tension developed between staff around the selection of the HLC role and further reports indicating the HLCs were not given the time by management to perform their role. Studies investigating an extended role of pharmacy support staff have identified a similar trend, in that more senior staff in the pharmacy are reluctant to delegate work and pharmacists reported a lack of confidence in the abilities of support staff.[59, 60] There was no mention in the reported studies of how this tension was managed in an attempt to successfully integrate the role of the HLC into the daily workflow.

Similarly, the learning climate, which refers to the motivation of staff to undertake new learning and the supportive environment to try new methods was identified as a positive implementation determinant. The enthusiasm of community pharmacy support staff to undertake further training and enhance their role in delivering services concurs with the limited literature in this area.[61] Despite this willingness to undertake further training, it was frequently mentioned that the inconvenience, time and cost investment for staff to undertake the HLC course was a barrier to achieving HLP accreditation. Although in the majority of cases, this was funded by local commissioners, the absence of a staff member from the pharmacy during the training period often caused an inconvenience and a cost implication to the pharmacy.

In more recent published reports, there was mention of the development of a HLC community of practice, which was used for peer-learning, seeking advice and obtaining information relevant to HLP accreditation.[36–38] This was identified as an enabler for HLP implementation and a potential resource for investment to support sustainability, however, to date there are no reports describing a wider adoption of this strategy.

### Individual characteristics of the CFIR

Table 4 provides an overview of the relevant *individual characteristics* constructs observed in the HLP literature.

The large majority of studies reported pharmacy staff enthusiasm and motivation to explore the opportunities, and undertake the different activities afforded by the HLP model. In two of the studies, it was reported that the local HLP launch event had contributed to individuals’ enthusiasm since these events were designed in such a way that supported the participation of attendees to offer their thoughts on the design and implementation of the initiative. The literature recognises that this approach can enhance buy-in of the stakeholders involved and promotes the decision to adopt an innovation.[62]

Community pharmacy staff tended to perceive that participation in the HLP initiative was a natural progression of their existing roles and fitted with their perceived values of community pharmacy. This observation is congruent with similar studies investigating pharmacy staff perceptions of introducing new services into the pharmacy.[63] The knowledge, confidence and skills gained by pharmacy staff completing the HLC course was frequently cited as a crucial factor and often a driver of implementation. However, it should be noted that the majority of studies investigating the views within community pharmacy were conducted with pharmacy teams who had made the decision to participate in the initiative and who were motivated to partake in the evaluative study. The views of pharmacy staff employed in pharmacies not involved in the HLP initiative is less well reported.

### Process

Table 4 provides an overview of the relevant *process* constructs observed in the HLP literature.

The *process* constructs identified in the literature were discussed with regards to their absence and subsequent detrimental effect on the implementation process. The lack of external change agents and poor communication to disseminate relevant HLP information was recognised as a barrier to HLP implementation and propagated feelings of professional isolation.

Although the introduction of the HLC role was widely considered to be a key enabler of HLP implementation in supporting staff training, influencing the attitudes and beliefs of their colleagues within the pharmacy and role-modelling good practice, their influence was largely limited to the pharmacy in which they worked. A lack of individuals who influenced or facilitated intervention decisions in a desirable direction, such as investing in enhancing wider public awareness of the HLP model was noted as a barrier in furthering the HLP agenda.

The description of the HLC role in these studies resonates strongly with the similar work describing the role of champions within community pharmacy in the implementation of new services. Pemberton *et al*.[64] drew on extant research, including studies from community pharmacy, in supporting the notion that innovation is facilitated and supported by innovation champions. Similarly, the research concluded that in order for innovation champions to succeed in promoting innovations in organisations, they needed both procedural and resource support, as well as social and cognitive support. The authors recognised that the influence of innovation champions came through social contacts, multiplied through the communities in which they participate, through the genuine esteem in which they are held. The authors recommended that developing a community around such champions makes practical sense for organisations and will potentially initiate further innovative practices.

Moreover, it has been recorded in a review of commissioning in community pharmacy services by the University of Manchester that the lack of an ‘external’ champion, such as a pharmaceutical advisor within the commissioning organisation, is likely to result in the discontinuation of a new community pharmacy service.[5]

### Reported outcomes

Table 5 provides a summary of the reported HLP outcomes observed in the literature.

In the majority of studies, the reported economic outcomes were based on staff perceptions rather than a robust cost-analysis of pre and post involvement in HLP involvement. This included staff perceptions that pharmacy income had increased through delivering more commissioned services in pharmacies working towards HLP accreditation.[35, 39, 46] Further studies revealed that pharmacy customers, reported a greater awareness of community pharmacy services, and a customer survey distributed nationally to customers who had used the services of an accredited HLP reported that 60% customers (n = 1034) would have otherwise visited their family doctor, which the authors argue, contributed to a potential cost saving to the NHS.[32] To date, there are no published reports that included a cost-analysis of HLP involvement; the commercial sensitivity of accessing service delivery and income data has been cited as a barrier to conducting an evaluation of this type.

Humanistic outcomes revealed the public’s acceptability of accredited HLP pharmacies as a new service provider to access health and well-being services, thereby increasing the potential options for the public to choose from. A recent systematic review revealed that whilst the literature most commonly reported that patient and public opinions about community services are positive, awareness of pharmacy services beyond medicines supply remains low.[65] Despite the finding of this present study indicating the public recognising HLP accredited pharmacies as a new option to access health services, the wider literature reports that patients still look to their physicians as their first point of access.[65]

The only published study to report service delivery data was the Portsmouth HLP pilot evaluation, which indicated HLP accredited pharmacies and pharmacies working towards accreditation successfully recruited and supported a greater number of customers to health and well-being services and medicines review services. However, other than demonstrating an increased proportion of smoking cessation clients abstaining from smoking after 12-weeks from the quit date, no other clinical data was reported. Furthermore, the report did not reveal whether or not these pharmacies were more active than others prior to becoming HLPs. Similarly, an unpublished report describing higher service provision in HLP accredited community pharmacies across one geographic location in England failed to report a baseline and therefore such observations were vulnerable to misinterpretations.[66]

### Summary of key findings

- This systematic review offers the first review of the published studies reporting on the HLP initiative and outcome data since its inception in 2009.
- Through adoption of the CFIR framework, this study has identified the implementation determinants relevant to the implementation of HLP into community pharmacy.
- A resonating issue emerged in that the absence of adopting an evidenced-based implementation process has limited the ability to capture meaningful outcome data. This has resulted in a lack of evidence to date to support sustainability and the failure to address many of the well cited barriers e.g. lack of awareness amongst patients, public and other healthcare professionals, weak support for future investment in resource for training and dissemination.

### Study strengths

It has recently been argued in the literature that application of implementation science in pharmacy practice is “long overdue” to promote the understanding or systematic testing of optimal ways to support implementation and sustainability.[67] In adopting the CFIR framework to analyse and interpret the reported implementation determinants of the HLP model, it has been possible to provide a description of reported innovation attributes that have been implicated in the implementation of HLP, namely the perceived *relative advantage* and *adaptability* of the model. Further, this study recognises the significant role of *inner and outer settings*, including the reported lack of public awareness and collaboration of local health care providers, and the organisational attributes that promoted or discouraged the implementation.

In recent times, researchers and commissioners have focussed on remuneration as the single most important factor in introducing innovation in community pharmacy with little consideration to other factors. However, it is now beginning to be acknowledged that implementation processes that attempt to address individual factors in isolation are unlikely to be successful. Change management research demonstrates that an understanding of social trends and forces affecting an organisation is essential to facilitate effective change.[68] This study provides further evidence of this phenomenon within the areas of pharmacy practice and public health.

This review also indicates how the implementation of the HLP model has failed to follow a series of recommended steps that would facilitate assessment and monitoring of success and sustainability. Crespo-Gonzalez et al. argues that innovative services should be well defined in relation to the target population, context, objectives, methodology outcomes and expected benefits. An impact assessment for key outcomes, patient and economic, via a pilot study, would also test for feasibility and a process evaluation would determine factors impacting on service success.[69] A recent case report of a pharmacist-led medication review service describes an implementation-effectiveness study. This demonstrates how the processual and contextual information and specific outcomes from the implementation procedure can be mapped out towards better understanding and assessment of the intervention in terms of clinical, humanistic and economic outcomes.[70]

### Study limitations

A limitation of this review is that of the twenty publications included in this study, the majority were conference abstracts (fourteen) which are often short reports of actual studies, presenting information that help practitioners and researchers to decide whether to attend a presentation. Some of the publications were written by authors of this study, which could be viewed as a limitation, although the quality assessment was conducted by at least one researcher who was independent of each publication included. Crucial study information is often missing in abstracts and suboptimal reports can impede the determination of the quality of the study and assessment of whether reliable conclusions can be drawn. This can often be a major obstacle to evidence synthesis. Guidance and checklists exist that can facilitate better reporting of interventions to allow for better description and assessment. These should be used as a standard to progress research and evidence further.[71] Moreover, there were no examples in the studies included where implementation literature was cited, thus findings were reported as observations and themes derived from the data, rather than adopting a framework approach to support interpretation. This posed a challenge for the researchers (ZN and HN) to interpret reported findings in relation to the CFIR domains and constructs, therefore analysis of included studies was carried out independently and any disagreements were discussed and resolved through consensus between both investigators and a third reviewer where necessary.

## Conclusion

This review has successfully collated the published literature relating to the Health Living Pharmacy model and provides detail of its reported evidence-base. With the exception of one study conducted by the research team in Portsmouth, the published studies do not include service delivery data from community pharmacies, but rather focus on the self-reported impact of introducing HLP. The majority of the literature consists of cross-sectional studies involving small samples of community pharmacy staff conducted in specific geographical areas of England. Recent changes to the community pharmacy contractual framework has seen the introduction of payments for meeting a set of defined quality criteria, one of which is designed around the HLP model. Further research is required to evaluate the impact these changes have made to community pharmacy. Most importantly, the need for robust outcome data is significant. Evidence for effectiveness will provide a powerful tool to bolster many of the implementation constructs, supporting future delivery and adoption of HLP.

The CFIR has demonstrated its value in describing the challenges of implementing the HLP model in community pharmacy. The findings from this review emphasise the importance of considering an evidence-based approach to the design and implementation of innovation within community pharmacy in order to enhance the successful integration of the innovation and its potential to contribute humanistic, economic and clinical outcomes.

## Supporting information

S1 Table Accreditation criteria Level 1 HLP[72]. (DOCX)

S2 Table Summary of JBI Qualitative Assessment and Review Instrument (QARI). (DOCX)

S3 Table MEDLINE using OV SP (English language only). (DOCX)

S4 Table PRISMA checklist. (DOC)
