# Correction: A systematic review of the outcome data supporting the Healthy Living Pharmacy concept and lessons from its implementation

**DOI:** 10.1371/journal.pone.0216804

**Published:** 2019-05-07

**Authors:** Zachariah Jamal Nazar, Hamde Nazar, Simon White, Paul Rutter

The following information is missing from the Funding statement: This article was funded by the Qatar National Library. The funder had no role in study design, data collection and analysis, decision to publish, or preparation of the manuscript.
