# Supplementary material for: A systematic review of the outcome data supporting the Healthy Living Pharmacy concept and lessons from its implementation
Source: PLoS One. 2019 Mar 12;14(3):e0213607. doi: 10.1371/journal.pone.0213607 (PMC6414028; doi:10.1371/journal.pone.0213607)
Supplement: S1 Table — (DOCX) [file pone.0213607.s001.docx]

**S1 Table: Accreditation criteria Level 1 HLP**

| **Criteria: A HLP** |
| --- |
| 1. Consistently delivers a range of health and well-being services to a high quality. Every interaction in the pharmacy should be seen as an opportunity for a health promoting intervention, ‘making every contact count’. |
| 1. Has achieved defined quality criteria requirements and met productivity targets linked to local health needs e.g. the number of successful Stop Smoking quits at 4 weeks |
| 1. Has a team that proactively promotes health and well-being and proactively offers brief advice in a range of health issues such as smoking, physical activity, sexual health, healthy eating and alcohol. |
| 1. Has a minimum of one Health Champion (also known as Healthy Living Champion), who has achieved the Understanding Health Improvement Level 2 Royal Society of Public Health award. The HLC is proactive in promoting health and wellbeing messages, signposts the public to appropriate services and enables and supports the team in demonstrating the ‘ethos’ of an HLP. |
| 1. Has premises that are fit for purpose for promoting health and wellbeing messages as well as delivering commissioned services. The consultation room should be equipped appropriately depending on the services offered. |
| 1. Engages with the local community and other health and social care professionals |
| 1. Is recognisable to the public through the display of the HLP logo |
| 1. Leadership training undertaken by an individual involved in a leadership or management position so that they can support the development of the pharmacy team and change from providing reactive to proactive health interventions. |
