# Supplementary material for: A systematic review of the outcome data supporting the Healthy Living Pharmacy concept and lessons from its implementation
Source: PLoS One. 2019 Mar 12;14(3):e0213607. doi: 10.1371/journal.pone.0213607 (PMC6414028; doi:10.1371/journal.pone.0213607)
Supplement: S2 Table — (DOCX) [file pone.0213607.s002.docx]

**S2 Table: Summary of JBI Qualitative Assessment and Review Instrument (QARI)**

| Study | Is there congruity between the stated philosophical perspective and the research methodology? | Is there congruity between the research methodology and the research question or objectives? | Is there congruity between the research methodology and the methods used to collect data? | Is there congruity between the research methodology and the representation and analysis of data? | Is there congruity between the research methodology and the interpretation of results? | Is there a statement locating the researcher culturally or theoretically? | Is the influence of the researcher on the researcher, and vice-versa addressed? | Are participants, and their voices adequately represented? | Is the research ethical or, is there evidence of ethical approval by approved body? | Do the conclusions drawn in the research report flow from the analysis, or interpretation of the data? | Score /10 |
| --- | --- | --- | --- | --- | --- | --- | --- | --- | --- | --- | --- |
| Kennington et al.(2013) | NO | YES | YES | YES | YES | NO | NO | NO | YES | YES | 6 |
| Nazar et al.  (2013) | UNCLEAR | YES | YES | NO | YES | NO | NO | NO | YES | YES | 5 |
| Kennington et al.(2013) | NO | NO | NO | NO | YES | NO | NO | NO | YES | YES | 3 |
| Brown et al. (2014) | UNCLEAR | YES | YES | YES | YES | NO | NO | YES | YES | YES | 7 |
| Rutter et al. (2014) | YES | YES | YES | YES | YES | NO | NO | YES | YES | YES | 8 |
| White et al.  (2014) | YES | YES | YES | NO | YES | NO | NO | NO | YES | YES | 6 |
| Donovan et al. (2014) | UNCLEAR | YES | YES | YES | YES | NO | NO | YES | YES | YES | 7 |
| Patel et al. (2014) | NO | NO | NO | NO | NO | NO | NO | NO | YES | YES | 2 |
| Shevket et al. (2015) | YES | YES | YES | YES | YES | NO | NO | NO | YES | YES | 7 |
| Firth et al. (2015) | UNCLEAR | YES | YES | YES | YES | NO | NO | YES | YES | YES | 7 |
| Donovan et al. (2015) | YES | YES | YES | YES | YES | NO | NO | YES | YES | YES | 8 |
| Mackridge et al.(2015) | YES | YES | YES | NO | NO | NO | NO | NO | YES | YES | 5 |
| Kayyali et al. (2016) | NO | NO | NO | NO | YES | NO | NO | NO | YES | YES | 3 |
| Nazar et al.  (2016) | YES | YES | YES | YES | YES | NO | NO | YES | YES | YES | 8 |
| White et al. (2016) | YES | YES | YES | YES | YES | NO | NO | YES | YES | YES | 8 |
| Cooper et al. (2017) | YES | YES | YES | YES | YES | NO | NO | YES | YES | YES | 8 |
| Nazar et al. (2017) | UNCLEAR | YES | YES | YES | YES | NO | NO | NO | YES | YES | 6 |
| Nazar et al. (2017) | YES | NO | YES | YES | NO | NO | NO | NO | YES | NO | 4 |
