# Supplementary material for: A systematic review of the outcome data supporting the Healthy Living Pharmacy concept and lessons from its implementation
Source: PLoS One. 2019 Mar 12;14(3):e0213607. doi: 10.1371/journal.pone.0213607 (PMC6414028; doi:10.1371/journal.pone.0213607)
Supplement: S3 Table — (DOCX) [file pone.0213607.s003.docx]

**S3 Table: MEDLINE using OV SP (English language only)**

| **Search** | **Query** | **Items found** |
| --- | --- | --- |
| #7 | Search #6 OR #4 OR #1 | 26 |
| #6 | Search #3 AND #5 | 3 |
| #5 | Search champion Filters: English | 4709 |
| #4 | Search #2 AND #3 | 29 |
| #3 | Search "healthy living" Filters: English | 1509 |
| #2 | Search pharmacy Filters: English | 362075 |
| #1 | Search “healthy living pharmacy” Filters: English | 8 |
